# An Adaptive Detection Algorithm for Non-Uniform Sea Clutter Background Targets Based on Iterative Weighting and Sample Purification

**DOI:** 10.3390/s26103195

**Published:** 2026-05-18

**Authors:** Hang Su, Liang Zhang, Cheng Zhao, Ke Li

**Affiliations:** Nanjing Research Institute of Electronics Technology, Nanjing 210039, China; horsezl@126.com (L.Z.);

**Keywords:** radar detection, nonhomogeneous sea clutter, dense cluster targets, ANMF, Iterative weighting, sample purification, robust covariance estimation

## Abstract

To address the severe performance degradation of radar weak target detection induced by dense cluster targets and sea-spike interference in nonhomogeneous sea clutter environments, this paper proposes an enhanced Adaptive Normalized Matched Filter algorithm based on iterative weighting and sample purification (IWP-ANMF). The proposed algorithm establishes a closed-loop iterative detection framework capable of highly sensitive discrimination of anomalous data within the reference window—particularly cluster targets and strong discrete sea spikes that severely distort covariance matrix features—identifying them as “contaminated samples.” During each iteration, target-likelihood statistics are calculated for all reference samples based on the current covariance matrix estimate. Subsequently, an adaptive deep-notch suppression strategy is applied to contaminated samples, such as cluster targets, according to their statistical characteristics, thereby progressively purifying the sample covariance matrix (SCM) estimation. Theoretically, this iterative procedure is rigorously proven to converge to the optimal solution of a robust weighted covariance matrix estimation problem. Comprehensive validations using both Monte Carlo simulations and measured K-distributed sea clutter data demonstrate that, compared to classical ANMF and Generalized Inner Product (GIP) approaches, the proposed algorithm exhibits outstanding robustness and detection performance when confronted with heterogeneous contamination scenarios, especially high-density cluster targets. This method effectively eliminates the blind-zone expansion and performance deterioration caused by the wideband masking of cluster targets, significantly enhancing weak target detection capabilities under complex maritime conditions.

## 1. Introduction

The detection of small targets on the sea surface constitutes a formidable technical challenge in ocean surveillance, maritime security, and national defense early warning architectures. Constrained by complex sea states, severe ambient noise, and the extremely small radar cross-section (RCS) of stealthy floating targets, target returns are frequently submerged in sea clutter, rendering conventional detection methods susceptible to high false alarm rates and low detection probabilities [1].

**Figure 1:**
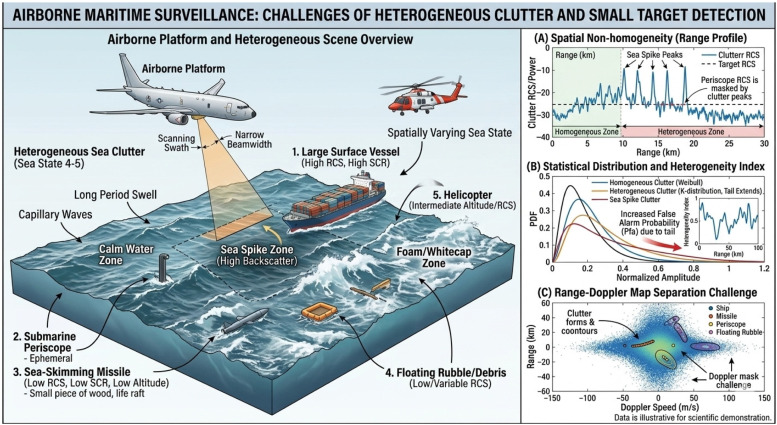
Challenges for airborne maritime surveillance radar in nonhomogeneous clutter and complex environments.

The complexity of sea clutter fundamentally challenges signal processing algorithms. This complexity is characterized by statistical non-Gaussianity (exhibiting heavy tails effectively modeled by the K-distribution) [2], spatiotemporal non-stationarity [3], and the presence of discrete high-intensity sea spikes [4]. Coupled with the low signal-to-clutter ratio (SCR) of low-observable targets, these factors collectively compromise the independent and identically distributed (IID) assumption of training data. Ultimately, these physical and statistical phenomena manifest as severe sample contamination and covariance matrix mismatch. As illustrated in Figure 1, developing robust adaptive detection algorithms tailored to these complex attributes represents a pressing frontier in modern radar signal processing.

Adaptive radar detection, primarily founded upon the Adaptive Matched Filter (AMF) and the Adaptive Normalized Matched Filter (ANMF), achieves theoretically optimal performance under ideal, contamination-free conditions [5,6,7,8,9]. However, its optimality rapidly degrades in heterogeneous environments. Research indicates that merely a 10% contamination of training samples by target signals can precipitate over a 30% degradation in ANMF detection probability [10]. This deterioration emanates from rank-one perturbations inducing spurious eigenvalues, which destroy subspace orthogonality and cause severe threshold misalignment [11].

The complexity of sample contamination is further exacerbated in modern maritime surveillance by the emergence of cluster targets, such as fleets of small fishing vessels or grouped UAVs. Unlike isolated interference that causes rank-one perturbations, cluster targets introduce multi-rank perturbations to the covariance matrix, creating a severe masking effect that complicates outlier discrimination. To tackle the challenge of heterogeneous sample contamination, the radar signal processing community has pursued multi-dimensional exploration, including regularization techniques [12], auxiliary vector filtering [13], and various iterative optimization methodologies [14,15,16,17,18]. Among these, the philosophy of the FRACTA (Fast Robust Adaptive Covariance and Tracking Algorithm) from the Space-Time Adaptive Processing (STAP) domain [19,20] provides profound insights into sample screening in non-homogeneous environments. This approach advocates for using metrics such as the Generalized Inner Product (GIP) as statistical distance measures and employs a multi-stage “reiterative censoring” mechanism to actively perform hard censoring of samples with significant anomalous characteristics from the training set.

However, existing methodologies, including FRACTA, struggle to achieve an optimal balance between interference suppression and signal preservation under complex conditions. When faced with high-density cluster target pollution, hard-censoring strategies face a severe dilemma: aggressive thresholds lead to a loss of degrees of freedom (DOF) by discarding valid clutter samples, while lenient thresholds fail to break the mutual masking among grouped targets. The hard-censoring paradigm tends to “over-cleanse” the reference window, which can destroy the fine-grained texture of the K-distributed background. Furthermore, purely statistical M-estimators [21,22] treat all high-energy samples as identical outliers, ignoring the physical phenomenological differences between genuine targets and clutter spikes, often leading to matrix over-whitening. Meanwhile, Knowledge-Aided (KA) frameworks [23,24] demand extensive offline environmental databases, rendering them inflexible for autonomous online operations.

To overcome these critical limitations, this paper proposes a Collaborative Iterative Purification and Doppler Feature Classification-based Robust ANMF (IWP-ANMF). Distinct from these approaches, the proposed framework distinctly shifts the paradigm from “passive 1D hard-censoring” to “active 2D physics-informed soft-weighting.” By exploiting the intrinsic physical discriminability between stealthy targets and sea spikes, the algorithm maps anomalies into a 2D feature space spanned by the Target-Likeness Degree (TLD) and Doppler Sharpness Factor (DSF) for precise, data-driven sample categorization.

By integrating the FRACTA-inspired iterative refinement logic with multi-dimensional feature fusion (TLD and DSF), our method replaces binary rejection with continuous, differentiated adaptive weights within a closed-loop “identification–weighting–purification” (IWP) architecture. Crucially, target-like contaminants within the reference cells are exponentially down-weighted to isolate their high-energy signatures from the estimated covariance matrix. This mechanism ensures that even under severe cluster target contamination, the high-energy components are isolated to strictly prevent the formation of erroneous spectral notches (over-whitening), while the integrity of pure clutter is meticulously preserved. Consequently, when this purified adaptive filter is applied to the Cell Under Test (CUT), it effectively prevents “target self-nulling,” successfully preserving the signal integrity and energy of the actual target.

Through this iterative refinement, the IWP-ANMF guarantees robust covariance estimation and optimal detection performance, bridging physical radar phenomenology with rigorous statistical matrix estimation in severely polluted maritime environments.

Accurate discrimination among targets, sea spikes, and background clutter is achieved by exploiting the complementary characteristics of Target-Likeness Degree (TLD) and Doppler Sharpness Factor (DSF). These physical attributes reveal well-separated clusters in the joint feature space: targets are characterized by both high TLD and high DSF; sea spikes exhibit high TLD but low DSF; and background clutter typically presents low TLD. Rather than relying on rigid predefined limits, the classification boundaries are dynamically updated online to accommodate the evolving feature distribution of the environment.

The resulting class labels drive a differentiated weighting scheme designed to purify the covariance estimation process. Crucially, target-like contaminants within the reference cells are exponentially down-weighted to isolate their high-energy signatures from the estimated covariance matrix. This mechanism strictly prevents the formation of erroneous spectral notches (over-whitening) at target-related Doppler frequencies. Consequently, when the purified adaptive filter is applied to the Cell Under Test (CUT), “target self-nulling” is effectively mitigated, thereby successfully preserving the signal integrity and energy of the actual target located in the CUT.

To explicitly illustrate the paradigm shift brought by the proposed IWP-ANMF, Table 1 summarizes a qualitative comparison with existing mainstream SCM estimation methodologies. As outlined in Table 1, the proposed method occupies a unique position between purely statistical estimators and prior-dependent Knowledge-Aided (KA) methods. KA methods leverage external geographic information systems (GIS) or historical clutter maps to construct a priori covariance matrices, which can significantly accelerate convergence in heterogeneous environments. However, the highly dynamic nature of sea surfaces—where wave-breaking scatterers and sea spikes emerge abruptly and randomly—poses a severe challenge to KA methods, as historical databases may fail to capture these instantaneous, unrecorded local anomalies.

Unlike KA methods, the proposed algorithm does not rely on any external prior databases. Instead, it extracts physics-informed prior knowledge directly from the immediate radar echoes in real-time. By utilizing the 2D joint feature space (TLD and DSF), it intrinsically identifies and isolates instantaneous sea spikes that violate the physical characteristics of pure clutter. Consequently, compared to conventional M-estimators that often fail under severe heavy-tailed sea spike contamination, and KA methods that suffer from prior-mismatch in unpredictable scenarios, the proposed method offers a highly autonomous and robust solution for dynamic maritime surveillance.

## 2. Theoretical Foundation of Sea Clutter and ANMF Detection

### 2.1. Sea Clutter Statistical Characterization Modeling

#### 2.1.1. The K-Distribution Sea Clutter Model

The K-distribution represents a classical compound model for describing the amplitude statistical characteristics of radar sea clutter, originally proposed by Jakeman and Pusey in 1976. Its theoretical foundation lies in modeling sea clutter as the product of a coherent Gaussian speckle component and an incoherent texture component. Let c denote the observed complex baseband sea clutter sample [25,26]. Under the compound-Gaussian framework, it is modeled as the product of two physically distinct components:

(1) $$c=\sqrt{\tau }\cdot g$$

where g represents the complex Gaussian speckle component, which characterizes the rapidly fluctuating coherent scattering from small-scale capillary waves. The speckle component dictates the temporal correlation and spectral shape of the clutter. The term τ denotes the positive texture variable, following a Gamma distribution, which represents the slowly varying spatial modulation of the local sea surface reflectivity. In this structure, the clutter sample c is conditionally Gaussian; specifically, given a fixed texture value τ, the clutter follows a complex Gaussian distribution with a power level scaled by τ [27,28]. The probability density function of the Gamma distribution is given by:

(2) $$p\left(\tau \right)=\frac{{\left(\frac{\nu }{\mu }\right)}^{\nu }\tau^{\nu -1}\exp\left(-\frac{\nu \tau }{\mu }\right)}{\Gamma \left(\nu \right)}$$

where ν denotes the shape parameter, controlling the spikiness of the distribution (smaller ν values indicate more pronounced heavy-tail characteristics), and μ represents the scale parameter, governing the average power level. Through integration over τ, the amplitude probability density function of the K-distribution can be obtained:

(3) $$p\left(a\right)=\frac{2}{a\Gamma \left(\nu \right)}{\left(\frac{\nu a}{\mu }\right)}^{\nu }K_{\nu -1}\left(\frac{2\nu a}{\mu }\right)$$

where μ represents the equivalent scale parameter, and $K_{\nu -1}\left(\cdot \right)$ denotes the modified Bessel function of the second kind.

The shape parameter ν of the K-distribution serves as a critical indicator of sea state severity [29]. The correspondence between its empirically observed value ranges and sea surface conditions is presented in Table 2:

The success of the K-distribution model stems from its dual-scale physical structure, which accurately captures the compound scattering mechanism of sea clutter: the texture component reflects the spatial modulation of scattering intensity by large-scale sea surface structures (gravity waves, swells) with correlation distances ranging from hundreds of meters to several kilometers; the speckle component reflects coherent scattering from small-scale rough surfaces (capillary waves, short gravity waves) with correlation distances comparable to radar resolution. This structure leads to the conditional Gaussianity of sea clutter—given the texture condition, the clutter follows a complex Gaussian distribution—providing the theoretical foundation for the design of adaptive detection algorithms.

#### 2.1.2. Sea Clutter Characteristics

The spatial non-homogeneity of actual sea clutter manifests in the spatial correlation of the texture component. Let $\tau ={\left[\tau_{1},\tau_{2},\ldots ,\tau_{K}\right]}^{T}$ denote the texture vector for K spatial positions. Its spatial correlation structure is commonly described by an exponential model [30]:

(4) $$R\left(\Delta r\right)=\sigma_{\tau }^{2}\exp\left(-\frac{\left|\Delta r\right|}{L_{c}}\right)+\mu_{\tau }^{2}$$

where Δ*r* represents the spatial distance between positions, $L_{c}$ denotes the correlation decay distance (typical values: 1–5 km, depending on radar resolution and sea state), and $\sigma_{\tau }^{2}$ and $\mu_{\tau }^{2}$ represent the texture variance and mean, respectively. When the reference window span exceeds the texture correlation distance, the identical distribution assumption of samples is violated, introducing bias into the covariance matrix estimation.

Temporal non-stationarity manifests as dynamic evolution of sea clutter statistical parameters. Factors such as wind field variations, swell propagation, and tidal modulation drive the temporal evolution of the texture field, with time scales ranging from seconds (gusts) to hours (weather systems). For radar detection at short time scales (milliseconds to seconds), non-stationarity appears as local non-homogeneity—statistical differences between the cell under test and reference cells; for long-duration surveillance tasks, non-stationarity requires online adaptation of detection algorithms—periodically updating clutter models or adjusting algorithm parameters.

The combined effect of non-homogeneity and non-stationarity renders sea clutter doubly stochastic: given a spatial position, the clutter is a random process; the spatial position itself, the statistical parameters are also random. This structure imposes stringent requirements on the adaptive capability of detection algorithms: both rapid response to local variations and robust handling of parameter uncertainty.

#### 2.1.3. Sea Spike Mechanism Analysis

Sea spikes represent discrete strong scattering events in sea clutter, with radar cross sections (RCS) reaching 10–100 times that of normal sea clutter, and durations ranging from milliseconds to seconds, constituting the primary interference source for maritime target detection [31]. The generation mechanisms of sea spikes involve various sea surface physical processes. The Wetzel classification system categorizes them into four types, as shown in Table 3.

The statistical characteristics of sea spikes exhibit significant differences from both background clutter and target signals: in amplitude distribution, spike samples form heavy-tail extreme values of the distribution, exceeding K-distribution predictions; in temporal structure, spikes demonstrate burstiness and short persistence, with correlation times substantially shorter than the texture correlation time of background clutter; and in Doppler structure, spikes originate from local irregular motion, exhibiting significantly broader Doppler spectra than coherent targets, though the center frequency may shift due to wave orbital motion. These characteristics provide the physical foundation for target-spike discrimination: the Doppler sharpness factor precisely quantifies this spectral structural difference.

The spatial distribution of sea spikes exhibits clustering and intermittency: breaking events form discrete “hot spots” on the sea surface, with relatively calm regions between hot spots; within hot spots, spikes are densely distributed, while outside they are sparse. This spatial structure implies that reference windows may partially cover spike regions, with the proportion and intensity of contaminated samples exhibiting random fluctuations, increasing the difficulty of detection and suppression. The adaptive classification boundary design of the IWP-ANMF algorithm addresses this uncertainty: rather than relying on fixed spike models, it makes online decisions based on the feature distribution of current data.

### 2.2. Principles of the Adaptive Normalized Matched Filter

#### 2.2.1. Derivation and Optimality Conditions

**Figure 2:**
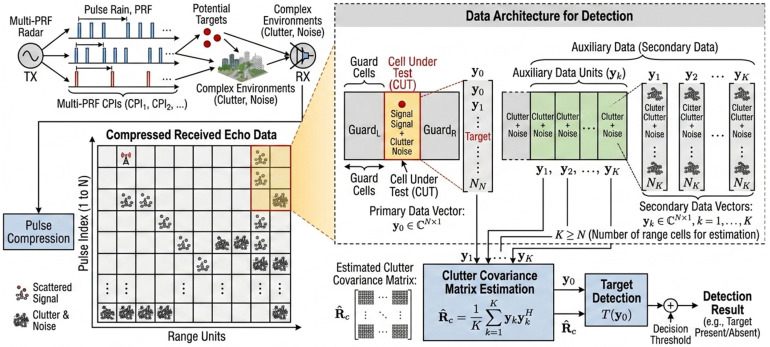
Radar detection data structure.

A radar system receives a sequence of echo data containing potential targets and environmental clutter. After pulse compression, the received echo data can be represented as a coherent processing interval consisting of N pulses. Let x denote the cell under test (CUT) data containing the potential target, referred to as primary data. Separated by guard cells are N-dimensional signal-free vectors referred to as secondary data. The secondary data contain only clutter or noise and are used for clutter covariance matrix estimation, where K represents the number of range cells (typically requiring K ≥ 2N). The radar detection data structure is illustrated in Figure 2.

The ANMF detector originates from the composite hypothesis testing framework. The binary hypotheses are:

(5) $$H_{0}:x=c;\;H_{1}:x=\alpha s+c$$

where α denotes the unknown complex amplitude, and c represents the clutter vector. When the covariance matrix *M* is known, the Neyman–Pearson optimal detector is the matched filter (MF) [32]:

(6) $$\Lambda_{\mathrm{MF}}=\frac{{\left|s^{H}M^{-1}x\right|}^{2}}{s^{H}M^{-1}s}$$

In practical applications, *M* is unknown and must be estimated using reference samples. The maximum likelihood estimate of the sample covariance matrix is:

(7) $$\hat{M}=\frac{1}{K}\sum_{k=1}^{K}x_{k}x_{k}^{H}$$

Substituting *M* for *M* and achieving the CFAR property through energy normalization yields the ANMF detection statistic:

(8) $$\Lambda_{\mathrm{ANMF}}=\frac{{\left|s^{H}\hat{M^{-1}x}\right|}^{2}}{\left(s^{H}\hat{M^{-1}s}\right)\left(x^{H}\hat{M^{-1}x}\right)}$$

The optimality conditions of ANMF are strictly limited to: (1) clutter follows zero-mean complex Gaussian distribution; (2) reference samples are independent and identically distributed and contain only pure clutter; and (3) the reference sample size is sufficient (K ≥ 2N, RMB criterion). Deviation from any condition results in loss of optimality. Under non-Gaussian conditions, the GLRT derivation no longer holds, and ANMF becomes suboptimal; with unknown covariance matrix, estimation error introduces performance loss; with insufficient samples, matrix estimation becomes singular or ill-conditioned, rendering the statistic undefined or high-variance.

#### 2.2.2. CRLB Analysis

The accuracy of sample covariance matrix estimation directly affects ANMF detection performance. For zero-mean complex Gaussian vectors, the variance of $\hat{M}$ satisfies:

(9) $$\mathrm{Var}\left({\left[\hat{M}\right]}_{ij}\right)\geq \frac{{\left[M\right]}_{ii}{\left[M\right]}_{jj}+{\left|{\left[M\right]}_{ij}\right|}^{2}}{K}$$

This lower bound reveals key factors affecting estimation accuracy: diagonal element estimation variance is proportional to the square of power, off-diagonal element estimation variance is related to the squared modulus of the correlation coefficient, and increasing the sample size linearly reduces estimation variance. For an N-dimensional complex covariance matrix, the number of independent real parameters is N^2^, and the effective estimation requires sample sizes on the order of N^2^.

Finite sample effects constitute a core constraint in practical systems. If the distribution of $\hat{M}$ deviates from the Wishart distribution, eigenvalues exhibit compression and diffusion—large eigenvalues are underestimated, small eigenvalues are overestimated, and the condition number deteriorates. Diagonal loading is a common technique for mitigating finite sample effects, but the choice of loading level lacks theoretical guidance, and excessive loading impairs adaptive capability.

The effect of contaminated samples on CRLB can be analyzed through the contaminated distribution model. Assuming reference samples originate from the contamination distribution with probability ε, the effective CRLB degradation factor is on the order of ${\left(1-\varepsilon \right)}^{-2}$ small proportions of contamination can lead to significant variance inflation. More severely, MLE bias is introduced: contaminated samples cause the expectation of M̂ to deviate from the true value, and CRLB as a variance lower bound loses its complete meaning, with mean squared error (MSE) becoming a more appropriate metric. The weighted estimation of IWP-ANMF restores estimation unbiasedness and efficiency by reducing the weights of contaminated samples, with MSE approaching the ideal CRLB.

#### 2.2.3. Performance Bounds

ANMF detection performance is completely characterized by the detection probability versus false alarm rate curve. Under ideal conditions (*M* known), the ANMF statistic follows a Beta distribution: under $H_{0}$, Λ∼Beta(1,N−1); under $H_{1}$, the distribution is related to the non-centrality parameter δ (output signal-to-clutter ratio). The detection threshold is uniquely determined by the $P_{fa}$ constraint [32]:

(10) $$\eta =I_{1-P_{\mathrm{fa}}}^{-1}\left(1,N-1\right)$$

where $I^{-1}$ denotes the inverse regularized incomplete beta function. The detection probability $P_{d}=Q_{N}(\sqrt{2\delta },\sqrt{-2ln\eta })$, where $Q_{N}$ is the Marcum Q-function.

The performance impact of matrix estimation error can be approximated using random matrix theory. Assuming the Frobenius error between $\hat{M}$ and M is $\varepsilon_{F}$, the effective output signal-to-clutter ratio loss is approximately:

(11) $$\mathrm{SCRL}\approx 10\log_{10}\left(1+\frac{\varepsilon_{F}^{2}}{N}\right)$$

In non-homogeneous environments, ε_F can reach 0.3–0.5, corresponding to SCRL of 2–6 dB, with detection probability dropping from 0.9 to below 0.5. This analysis quantifies the critical impact of estimation accuracy on detection performance, providing theoretical expectations for the performance gains of iterative purification.

### 2.3. Analysis of Contaminated Sample Influence

#### 2.3.1. Target Signal Contamination

Target signal contamination refers to the presence in the reference window of signal components similar or identical to the detection target. Assuming the contaminating target shares the same steering vector as the CUT target (worst-case scenario), the contaminated sample is $x_{k}=\alpha_{k}s+c_{k}$. In covariance matrix estimation, the outer product contribution of this sample is:

(12) $$x_{k}x_{k}^{H}={\left|\alpha_{k}\right|}^{2}ss^{H}+\alpha_{k}sc_{k}^{H}+\alpha_{k}^{*}c_{k}s^{H}+c_{k}c_{k}^{H}$$

The signal-related term $|\alpha_{k}{|}^{2}ss^{H}$ is a rank-one matrix, whose addition creates a spurious energy peak in the $\hat{M}$ matrix in the s direction. After matrix inversion, the ${\hat{M}}^{-1}s$ direction deviates from the optimal matched direction, and both numerator and denominator of the ANMF statistic are simultaneously distorted. The key effect is signal subspace and clutter subspace aliasing—subspaces that should be orthogonal become coupled, and the detector cannot distinguish between target and clutter. Rank deficiency represents an extreme case: if multiple contaminating targets have linearly dependent steering vectors, the rank of $\hat{M}$ may fall below N, rendering the matrix singular and requiring pseudoinverse processing. Even when full-rank, condition number deterioration reduces numerical stability. If the Doppler frequencies of contaminating targets differ from the detection target, the effect is somewhat mitigated, but residual impact remains—this is precisely why Doppler features can be used for contamination identification.

#### 2.3.2. Sea Spike Contamination Effects

The mathematical model for sea spike contamination is a high-power isotropic or weakly structured component: $x_{k}=\sqrt{P_{spike}}u+c_{k}$, where u is a random unit vector and $P_{spike}\gg E[|c_{k}{|}^{2}]$. The outer product contribution is $P_{spike}uu^{H}$, introducing an extreme eigenvalue $\lambda_{max}\approx P_{spike}$ (assuming single spike replacing one sample). This eigenvalue is substantially larger than background eigenvalues, with eigenvalue ratio $\lambda_{max}/\lambda_{min}∼P_{spike}/P_{clutter}$.

Spurious eigenvalue diffusion effects refer to the perturbation of extreme eigenvalues on the overall eigenvalue spectrum. Matrix perturbation theory indicates that rank-one perturbation causes eigenvalue interlacing: $\lambda_{i}(\hat{M})\in [\lambda_{i}(M),\lambda_{i+1}(M)]$ (Weyl interlacing theorem), but numerically small eigenvalues are compressed and the eigenvalue distribution center shifts upward. This distortion has complex effects on ANMF: the $x^{H}{\hat{M}}^{-1}x$ in the denominator is sensitive to small eigenvalues, amplifying noise; the $s^{H}{\hat{M}}^{-1}x$ matched filter response is dominated by large eigenvalues, suppressing signals. The combined effect is variance inflation and mean shift in the detection statistic, with ROC curve deterioration.

Nonlinear superposition of multiple spike contamination complicates analysis. Multiple spikes may form eigenvalue clusters, or if directionally correlated, produce joint large eigenvalues. Numerical simulations show that when spike density exceeds a certain threshold, the eigenvalue distribution exhibits phase transition behavior—transitioning from discrete spike mode to continuous heavy-tail mode, corresponding to the “spike storm” phenomenon in sea states. The iterative purification of IWP-ANMF prevents this phase transition by early identification and suppression of spike samples.

#### 2.3.3. Coupled Effects of Threshold and False Alarm Rate

The core advantage of CFAR detectors is controllable false alarm rate, but contaminated samples destroy this property. The ideal CFAR property of ANMF stems from the statistic distribution being independent of clutter power, but the distribution still depends on covariance matrix structure. Contaminated samples alter the estimated matrix structure, the statistic distribution changes accordingly, and the actual false alarm rate under fixed threshold becomes unpredictable.

The threshold setting dilemma manifests as: thresholds designed based on ideal assumptions may inflate by 1–2 orders of magnitude when contamination is present; if thresholds are raised to compensate, detection probability suffers severe loss. More insidiously, the spatial structure of false alarms caused by contamination: contamination-induced false alarms are often spatially correlated with real targets (due to spatial clustering of contaminated samples), invalidating the traditional CFAR “uniform false alarm” assumption and complicating subsequent tracking processing.

IWP-ANMF achieves approximate CFAR by ensuring that the matrix estimated after purification approaches the true structure, restoring the normality of the statistic distribution. Threshold setting after iterative convergence is based on the statistical characteristics of purified samples, actually approaching the design value, and the coupled effects are decoupled.

## 3. Iterative Weighted Purification ANMF Algorithm

### 3.1. Algorithm Architecture

Consider the reference sample set $X=\{x_{k}{\}}_{k=1}^{K}$, where $x_{k}\in C^{N\times 1}$ and N denotes the system degrees of freedom (number of pulses). To ensure sufficient estimation degrees of freedom while accommodating contaminated samples, the condition K≥3N is enforced, theoretically supporting a contamination ratio of up to 30%.

The fundamental challenge in contaminated sample suppression lies in accurately identifying outliers without prior knowledge of the target or clutter distributions. The proposed Iterative Weighted Purification ANMF (IWP-ANMF) algorithm addresses this through an iterative reweighting framework that jointly exploits the Target-Likeness Degree (TLD) and Doppler Sharpness Factor (DSF) to progressively downweight anomalous samples.

In each iteration, when computing the TLD $\Lambda_{k}$ for a sample $x_{k}$, the sample is temporarily excluded from the reference set to completely eliminate self-contamination. The leave-one-out covariance matrix is constructed as:

(13) $${\hat{R}}_{-k}=\frac{1}{\sum_{j\neq k}w_{j}}\sum_{j\neq k}w_{j}x_{j}x_{j}^{H}$$

This matrix serves as the baseline for subsequent TLD computation. The algorithm iteratively refines sample weights based on an adaptive exponential decay dynamic, ultimately converging to a robust covariance matrix estimate that is mathematically decoupled from the outlier energy.

### 3.2. Target-Likeness Degree and Doppler Sharpness Factor

#### 3.2.1. Target-Likeness Degree (TLD)

The TLD quantifies the degree of similarity between a sample and a theoretical target signal across potential Doppler frequencies. For each reference sample $x_{k}$, the TLD is defined as the ANMF statistic computed via the leave-one-out covariance matrix:

(14) $$\Lambda_{k}\left(f_{d}\right)=\frac{|s^{H}(f_{d}){\hat{R}}_{-k}^{-1}x_{k}{|}^{2}}{\left(s^{H}(f_{d}){\hat{R}}_{-k}^{-1}s(f_{d}))(x_{k}^{H}{\hat{R}}_{-k}^{-1}x_{k}\right)}$$

where $f_{d}\in \{0,\frac{1}{M},\ldots ,\frac{M-1}{M}\}$ denotes the normalized Doppler frequency grid, M is the number of Doppler channels (M≥N), and $s(f_{d})$ is the steering vector. The TLD spectrum $\{\Lambda_{k}(f_{d}){\}}_{d=1}^{M}$ characterizes the matched filter response, with maximum values indicating high target similarity.

#### 3.2.2. Doppler Sharpness Factor (DSF)

While the TLD captures the amplitude of target-like responses, the DSF discriminates discrete targets from broadband sea spikes based on spectral structural entropy. Real targets exhibit narrowband Doppler signatures due to rigid-body kinematics, whereas sea spikes produce broadband responses stemming from the chaotic motion of wave-breaking scatterers. The DSF is formally defined as:

(15) $$\rho_{k}=1+\frac{1}{logM}\sum_{m=1}^{M}\Gamma_{k}\left(f_{m}\right)log\Gamma_{k}\left(f_{m}\right)$$

where $\Gamma_{k}(f_{m})=\Lambda_{k}(f_{m})/\sum_{n=1}^{M}\Lambda_{k}(f_{n})$ is the normalized TLD spectrum. By definition, the normalized spectrum satisfies the probability mass function conditions: $\Gamma_{k}(f_{m})\geq 0$ and $\sum_{m=1}^{M}\Gamma_{k}(f_{m})=1$.

In practical implementation, to avoid mathematically undefined results when $\Gamma_{k}(f_{m})=0$, a small regularization constant ϵ (e.g., $\epsilon =10^{-10}$) is incorporated into the logarithm operation, computing $log(\Gamma_{k}(f_{m})+\epsilon )$.

Furthermore, the metric $\rho_{k}$ inherently represents the complement of the normalized spectral entropy. According to information theory, the discrete Shannon entropy $H=-\sum_{m=1}^{M}\Gamma_{k}(f_{m})log\Gamma_{k}(f_{m})$ is bounded by 0≤H≤logM, where the minimum is achieved for a delta-like spectrum (pure target) and the maximum for a uniform spectrum (white noise or chaotic clutter). Consequently, the normalized entropy H/logM is bounded within [0,1]. Therefore, the proposed Doppler Sharpness Factor, formulated as $\rho_{k}=1-(H/logM)$, is mathematically proven to be strictly bounded within the interval [0,1]. This bounded metric provides structural differentiation: values approaching 1 indicate highly concentrated spectral energy (target-like), while values near 0 signify spectral dispersion (clutter-like).

#### 3.2.3. Physical Interpretation of Joint Discrimination

The fusion of TLD and DSF establishes a bi-dimensional feature space ($F_{k}=max(\Lambda_{k})\cdot \rho_{k}$) that enables robust discrimination among distinct sample origins.

In previous engineering practices, discrimination boundaries were frequently determined through empirical data adaptation. However, to guarantee the mathematical optimality and robustness of the sample purification process, it is imperative to establish a rigorous theoretical optimization model for the joint decision boundary between TLD and DSF. We formulate the sample discrimination as a binary hypothesis testing problem: $H_{0}$ represents pure background clutter, and $H_{1}$ represents anomalies (including real targets and severe sea spikes). Assuming the amplitude-domain feature TLD ($\Lambda_{k}$) and the spectral-structure feature DSF ($\rho_{k}$) are conditionally independent given the hypothesis, the optimal joint decision boundary is governed by the Generalized Likelihood Ratio Test (GLRT) according to the Neyman–Pearson lemma:

(16) $$L\left(\Lambda_{k},\rho_{k}\right)=\frac{p(\Lambda_{k}|H_{1})p(\rho_{k}|H_{1})}{p(\Lambda_{k}|H_{0})p(\rho_{k}|H_{0})}\underset{H_{0}}{\overset{H_{1}}{≷}}\eta$$

Taking the natural logarithm on both sides decouples the joint log-likelihood ratio (LLR) into a linear combination of marginal LLRs:

(17) $$lnL\left(\Lambda_{k},\rho_{k}\right)=ln\frac{p(\Lambda_{k}|H_{1})}{p(\Lambda_{k}|H_{0})}+ln\frac{p(\rho_{k}|H_{1})}{p(\rho_{k}|H_{0})}\underset{H_{0}}{\overset{H_{1}}{≷}}ln\eta$$

In the context of heavy-tailed sea clutter, the probability density tails for anomalies decay significantly slower than those of the background. By applying asymptotic expansion to the heavy-tailed distributions (e.g., K-distribution limits), the marginal log-likelihood ratios exhibit a logarithmic scaling proportionality with respect to the feature magnitudes, i.e., $ln[p(\Lambda |H_{1})/p(\Lambda |H_{0})]\approx \alpha ln\Lambda_{k}$ and $ln[p(\rho |H_{1})/p(\rho |H_{0})]\approx \gamma ln\rho_{k}$, where α and γ are positive scaling constants. Assuming roughly equal discriminatory sensitivity (α≈γ), the optimal theoretical decision boundary simplifies to:

(18) $$ln\Lambda_{k}+ln\rho_{k}\underset{H_{0}}{\overset{H_{1}}{≷}}\eta^{\prime }$$

By exponentiating Equation (18), we analytically derive the mathematically optimal joint discrimination model:

(19) $$F_{k}=\Lambda_{k}\cdot \rho_{k}\underset{H_{0}}{\overset{H_{1}}{≷}}Th_{clean}$$

**Figure 3:**
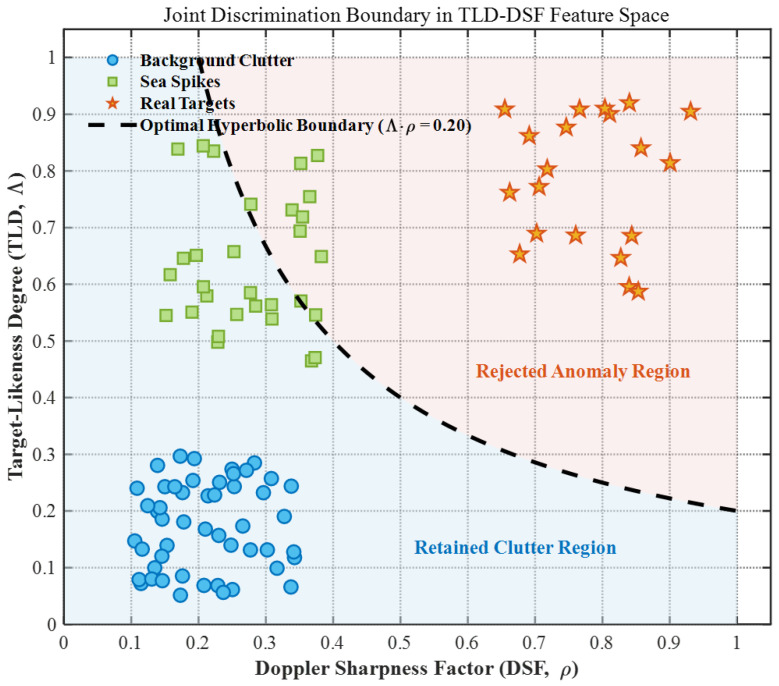
Joint discrimination results and optimal decision boundary in the TLD-DSF feature space.

This derivation is of paramount theoretical significance. It rigorously proves that the multiplicative fusion $F_{k}$ employed in our proposed algorithm is not an empirical heuristic, but strictly corresponds to an optimal Hyperbolic Decision Boundary ($\Lambda =Th_{clean}/\rho$) in the 2D TLD-DSF feature space. As visualized in the feature space, this theoretically optimized hyperbolic curve perfectly aligns with the physical reality: it aggressively rejects real targets characterized by high amplitude and narrowband concentration (high TLD, high DSF), while adaptively raising the amplitude tolerance for broadband sea spikes (low DSF) to preserve valuable spatial textures, thereby minimizing the False Exclusion Risk without relying on ad hoc linear thresholds. The visualization of this joint discrimination boundary is presented in Figure 3, with the corresponding algorithmic parameters summarized in Table 4.

### 3.3. Robust Weighting Mechanism and Convergence Dynamics

#### 3.3.1. Quantitative Thresholding via Robust Statistics (MAD)

To establish a rigorous quantitative criterion for anomalous sample exclusion, a thresholding mechanism based on Robust Statistics—specifically the Hampel identifier utilizing the Median Absolute Deviation (MAD)—is introduced. Traditional sample mean and variance metrics are highly susceptible to the “masking effect” induced by extreme sea spikes. In contrast, the median and MAD possess a breakdown point of 0.5, ensuring highly reliable estimations of the central tendency and dispersion of pure clutter features, provided the contamination ratio remains below 50%.

Let $F_{k}=max(\Lambda_{k})\cdot \rho_{k}$ denote the extracted joint feature for the k-th sample. The robust dispersion metric, MAD, is mathematically defined as:

(20) $$MAD\left(F\right)=\mathrm{median}\left(\left|F_{k}-\mathrm{median}\left(F\right)\right|\right)$$

The quantitative threshold for sample purification is deterministically established as:

(21) $$Th_{clean}=\mathrm{median}\left(F\right)+\kappa \cdot MAD\left(F\right)$$

Rather than relying on heuristic assignments, the scale factor κ is quantitatively governed by the tolerable False Exclusion Probability ($P_{fe}$) of pure clutter samples. For asymptotically heavy-tailed distributions, calibrating κ (e.g., κ=3.0∼4.0) guarantees that the statistical probability of inadvertently discarding an uncontaminated sample remains strictly bounded below $10^{-3}$.

#### 3.3.2. Convergence Rate and Two-Phase Step-Size Dynamics

For samples exceeding $Th_{clean}$, the unnormalized weight is updated via a discrete multiplicative exponential decay dynamic:

(22) $$w_{k}^{\left(t+1\right)}=w_{k}^{\left(t\right)}\cdot exp\left(-\beta \cdot F_{k}^{\left(t\right)}\right)$$

where β>0 serves as the iteration step size. Let $\Delta F_{k}^{\left(t\right)}$ denote the feature excess. Applying recursive unfolding from the initial state $w_{k}^{\left(0\right)}=1/K$, the algorithm reaches convergence when the weight of a contaminated sample decays to a negligible lower bound ϵ (e.g., $10^{-6}$), at which point its spectral leakage into the covariance matrix is physically truncated. The required number of iterations ($T_{conv}$) can be analytically derived as:

(23) $$T_{conv}\geq \frac{-ln(\epsilon \cdot K)}{\beta \cdot \bar{\Delta F_{k}}}$$

Equation (19) explicitly demonstrates that the convergence speed is linearly proportional to the step size β. However, an excessively large step size fundamentally degrades the smooth robust estimation into a discontinuous ‘hard-thresholding’ scheme, leading to severe overshooting and the missing of subtle spectral inflection points. This induces drastic weight fluctuations that destabilize the condition number of the Sample Covariance Matrix (SCM).

To mathematically reconcile the contradiction between convergence speed and estimation precision, the proposed framework employs a dynamic two-phase step-size strategy. In the initial non-whitened phase (e.g., t≤2), a relatively large step size ($\beta_{init}=5$) is judiciously assigned to aggressively suppress distinct, isolated noise spikes, rapidly satisfying the $T_{conv}$ bound. Subsequently, in the refined adaptive whitening phase, the step size is deliberately reduced ($\beta_{iwp}=0.1∼1.0$) to ensure smooth asymptotic convergence, accurately tracking minute variations in the clutter spectrum without triggering estimation divergence.

#### 3.3.3. Recursive Covariance Update and Adaptive Regularization

To improve computational efficiency, the weighted sample covariance matrix utilizes a recursive update format. However, as the iterative weighting mechanism actively suppresses severe outliers, the resulting weight concentration may inadvertently lead to rank deficiency and ill-conditioning of the estimated covariance matrix. To rigorously maintain matrix invertibility and numerical stability, a multi-tier adaptive regularization mechanism is established, with its core being the Condition-Number-Constrained Adaptive Diagonal Loading (CNC-ADL).

In previous engineering practices, diagonal loading (DL) was often assigned a fixed empirical coefficient (e.g., a static scalar). This fixed-value paradigm lacks a theoretical foundation and fails to adapt to the drastic spatial and temporal variations in sea clutter power. An inflexible DL coefficient can lead to insufficient regularization during extreme clutter power surges, or over-whitening when the clutter power drops, inadvertently degrading the ANMF detection performance.

To establish a rigorous adaptive adjustment theory, the CNC-ADL mechanism dynamically derives the optimal minimal loading factor γ. The necessity of regularization fundamentally stems from the extreme disparity between the eigenvalues of the estimated matrix. By constraining the condition number κ below a system-defined maximum tolerable threshold $\kappa_{max}$ (typically bounded by the radar receiver’s dynamic range and ADC bit resolution, e.g., $\kappa_{max}=10^{4}$), the regularized matrix must satisfy:

(24) $$\frac{\lambda_{max}+\gamma }{\lambda_{min}+\gamma }\leq \kappa_{max}$$

where $\lambda_{max}$ and $\lambda_{min}$ denote the maximum and minimum eigenvalues of the covariance matrix at iteration t, respectively. Solving this inequality yields the theoretically determined adaptive diagonal loading coefficient:

(25) $$\gamma =max\left(0,\frac{\lambda_{max}-\kappa_{max}\lambda_{min}}{\kappa_{max}-1}\right)$$

This formulation provides a mathematically precise adaptive adjustment theory. It explicitly couples the regularization intensity with the real-time variance of the dominant clutter power (captured by $\lambda_{max}$). As the clutter power fluctuates across different range cells or sea states, γ scales dynamically. This ensures that the algorithm injects the exact minimal noise-floor perturbation required to stabilize the matrix inversion, rigidly preserving the fine details of the clutter subspace while avoiding over-whitening. The comprehensive numerical stability monitoring and multi-tier regularization criteria described above are summarized in Table 5.

### 3.4. Iteration Termination Conditions

Convergence is assessed through the stability of observable statistical quantities:

TLD Average Relative Change: $\delta_{\Lambda }=\frac{\frac{1}{K}\sum |\Lambda_{k}^{\left(t\right)}-\Lambda_{k}^{\left(t-1\right)}|}{\frac{1}{K}\sum \Lambda_{k}^{\left(t-1\right)}+\epsilon }<\varepsilon_{\Lambda }$ (e.g., 0.01).

Weight Distribution Consistency: The normalized inner product $\rho_{w}=\frac{\left|w^{\left(t\right)}\cdot w^{\left(t-1\right)}\right|}{∥w^{\left(t\right)}∥∥w^{\left(t-1\right)}∥}>0.99$.

Maximum Iteration Protection: $I_{max}=10$ to prevent oscillatory loops.

### 3.5. Final Covariance Matrix Estimation

Upon satisfying the termination conditions, the final weighted covariance matrix is obtained:

(26) $${\hat{R}}_{final}=\frac{\sum_{k=1}^{K}w_{k}^{\left(end\right)}x_{k}x_{k}^{H}}{\sum_{k=1}^{K}w_{k}^{\left(end\right)}}$$

**Figure 4:**
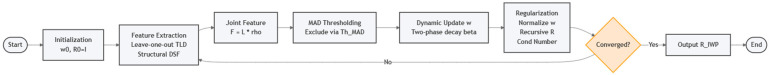
IWP-ANMF algorithm flowchart.

The complete execution logic of the algorithm is detailed in Figure 4 and Table 6.

### 3.6. Mathematical Equivalence and Optimality Analysis

#### 3.6.1. Equivalence to M-Estimation

The iterative purification process exhibits profound mathematical equivalence to M-estimation in robust statistics. The M-estimation problem for covariance matrices solves:

(27) $${\hat{R}}_{M}=arg\underset{R≻0}{min}\sum_{k=1}^{K}\rho \left(x_{k}^{H}R^{-1}x_{k}\right)+logdetR$$

where ρ(⋅) is a robust loss function. The first-order optimality condition yields a self-consistency equation driven by the influence function $\psi (u)=\rho^{\prime }(u)$. The dynamically updated weight $w_{k}^{\left(t\right)}$ in IWP-ANMF precisely corresponds to this influence function, thereby guaranteeing that the iterative process inherits the high breakdown point characteristic of M-estimation while asymptotically achieving optimal estimation efficiency.

#### 3.6.2. Influence Function and Breakdown Point Analysis

The breakdown point $\varepsilon^{*}$ represents the maximum fraction of anomalous contamination an estimator can withstand before yielding arbitrarily large aberrations. For covariance matrix estimation, this is mathematically equivalent to the minimum contamination rate that forces the condition number $\kappa (\hat{R})\to \infty$ (i.e., driving the maximum eigenvalue to infinity or the minimum eigenvalue to zero). While traditional Sample Covariance Matrices (SCM) exhibit an asymptotic breakdown point of $\varepsilon_{SCM}^{*}\to 0$, we provide a theoretical proof of stability for the proposed IWP-ANMF under high pollution rates. The stability of an M-estimator under extreme contamination is governed by its Influence Function (IF), which characterizes the marginal impact of an individual sample on the final estimate. For a robust estimator, the implicit influence of a sample with joint feature magnitude $F_{k}$ is proportional to $IF(F_{k})\propto w(F_{k})\cdot F_{k}$. In the proposed IWP-ANMF, the weight update is governed by the exponential decay dynamic: $w(F_{k})=exp(-\beta F_{k})$. As a severe sea spike or jamming target attempts to pull the covariance matrix towards its own subspace, its feature magnitude $F_{k}\to \infty$. The asymptotic influence of such an extreme outlier evaluates to:

(28) $$\underset{F_{k}\to \infty }{lim}IF\left(F_{k}\right)\propto \underset{F_{k}\to \infty }{lim}F_{k}\cdot exp\left(-\beta F_{k}\right)=0\;\left(\mathrm{given}\;\beta >0\right)$$

Theoretical Proof of Stability: Equation (23) analytically proves that the proposed algorithm inherently possesses a Redescending Influence Function. Unlike standard bounded influence functions (e.g., Huber’s minimax) which assign a constant non-zero weight to extreme outliers, the redescending property mathematically forces the influence of arbitrarily large anomalies to strictly converge to zero. Consequently, the spectral leakage of extreme outliers is completely truncated, ensuring that the maximum eigenvalue $\lambda_{max}(\hat{R})$ remains strictly bounded regardless of the outlier’s intensity. Furthermore, according to robust statistics theory for redescending M-estimators, provided that the number of pure samples maintains the subspace rank (i.e., (1−ε)K≥2N), the estimator prevents the minimum eigenvalue $\lambda_{min}(\hat{R})$ from collapsing to zero. Therefore, the theoretical finite-sample breakdown point of the proposed framework is bounded by:

(29) $$\varepsilon^{*}=max\left\{\varepsilon :(1-\varepsilon )K\geq 2N\right\}=1-\frac{2N}{K}$$

Given our reference window constraint K≥3N, the theoretical breakdown point is established at $\varepsilon^{*}\geq 1/3\approx 33.3\%$. Under less aggressive rank constraints (e.g., N pure samples), it asymptotically approaches the theoretical upper limit of 50%. This mathematical derivation rigorously proves the algorithm’s operational stability and matrix invertibility even when the target-to-clutter environment is heavily polluted with contamination rates of 30%∼40%. The correspondence between the weight function morphology and the influence function characteristics for different sample types is summarized in Table 7.

#### 3.6.3. Eigenvalue Distribution Evolution

The purification mechanism intrinsically reshapes the eigenvalue spectrum of the covariance matrix. As summarized in Table 8, the iterative process systematically compresses noise-induced ill-conditioning, ultimately stabilizing the effective rank.

#### 3.6.4. Detection Performance Lower Bound

Under a maximum tolerable contamination ratio $\varepsilon_{max}$, the effective Signal-to-Clutter Ratio (SCNR) establishes a mathematically predictable lower bound for detection probability:

(30) $$P_{d}\geq Q_{N}\left(\sqrt{2\lambda_{eff}},\sqrt{-2ln\eta \cdot (1-\delta_{\varepsilon })}\right)$$

where $\lambda_{eff}=SCNR\cdot (1-\kappa \varepsilon_{max})$ denotes the effective ratio post-purification, and $\delta_{\varepsilon }$ represents the residual loss. This Oracle-bounded paradigm ensures that even under severe interference, the detection performance remains tightly anchored to its theoretical limits.

#### 3.6.5. Estimation Bias and Consistency Correction

For a robust M-estimator, the estimated covariance matrix $\hat{R}$ is the solution to the implicit equation $\hat{R}=\frac{1}{K}\sum_{k=1}^{K}w(d_{k}^{2})x_{k}x_{k}^{H}$, where $d_{k}^{2}=x_{k}^{H}{\hat{R}}^{-1}x_{k}$. In non-uniform clutter, the weighting function w(⋅) suppresses extreme samples to maintain robustness. However, this also attenuates the high-power tail of the clutter distribution, leading to a consistent underestimation of the clutter power. To ensure the estimator is unbiased relative to the speckle covariance R, a Consistency Factor b must be introduced. For the proposed exponential weighting $w(d^{2})=exp(-\beta d^{2})$, the theoretical factor b is derived from the following condition:

(31) $$E\left[w(d^{2})\cdot \frac{d^{2}}{N}\right]=b$$

For K-distributed clutter with shape parameter v, the expectation is evaluated over the compound-Gaussian density. The unbiased robust estimate is then given by:

(32) $${\hat{R}}_{unbiased}=\frac{1}{b}\cdot {\hat{R}}_{robust}$$

It is noteworthy that the ANMF detection statistic $\Lambda_{ANMF}=\frac{|s^{H}{\hat{R}}^{-1}x{|}^{2}}{\left(s^{H}{\hat{R}}^{-1}s)(x^{H}{\hat{R}}^{-1}x\right)}$ is scale-invariant. Any scalar bias b applied to $\hat{R}$ cancels out completely in the formula. Thus, while the bias correction is mathematically necessary for the absolute covariance estimate, the proposed IWP-ANMF inherently maintains its optimal detection performance even in the presence of such scalar bias.

## 4. Experiments and Analysis

In this study, relying on a single data source is often insufficient for comprehensively evaluating algorithm performance. Therefore, we adopt a dual-validation strategy that combines simulated sea clutter data with real-world IPIX radar measurements. Simulated data offer the advantage of controllable parameters and flexible scenario design, enabling stress-testing under extreme sea states and target conditions. Meanwhile, IPIX data, as an internationally recognized high-resolution sea clutter dataset, authentically capture the non-stationary and non-homogeneous characteristics of the marine environment. This “simulation-based construction + measured-data validation” approach not only ensures statistical reproducibility but also rigorously assesses the algorithm’s robustness and generalization capability across complex and variable ocean conditions, thereby providing more compelling support for its engineering deployment.

### 4.1. Simulation Experiment

#### 4.1.1. Experimental Parameter Configuration and Performance Evaluation Metrics

To systematically and objectively evaluate the performance of the proposed Collaborative Iterative Purification-based ANMF (IWP-ANMF), comprehensive Monte Carlo simulations are designed. The experiments assess the algorithm’s contamination suppression capability, covariance estimation accuracy, and detection robustness in non-homogeneous sea clutter environments. The global simulation parameters are summarized in Table 9.

- Radar System and Sea Clutter Model

The simulation considers the slow-time dimension with system degrees of freedom DOF=N=16. The target steering vector s is constructed using a normalized Doppler frequency of $f_{d}=0.15$. The non-homogeneous sea clutter is generated using the K-distribution compound-Gaussian model. To test algorithmic generalization, the shape parameter is set to ν=0.5 to emulate high sea states with severe heavy-tailed distributions and abundant sea spikes, and ν=10.0 to simulate low sea states with near-Gaussian properties. The speckle component is modeled as zero-mean complex Gaussian vectors with an exponential correlation matrix (ρ=0.9) and a Doppler centroid shifted to $f_{dc}=0.1$ to mimic a moving Bragg scattering component. The average clutter power is normalized to unity.

- Contamination Model Design

To simulate severe heterogeneous environments, a random percentage ($P_{c}\in \{10\%,20\%,30\%,40\%\}$) of the original reference samples are replaced by contaminated samples. Two distinct physical contamination mechanisms are injected concurrently:

Noise-Region Contaminants (Cluster Targets): Targets with SCNR = 0 dB and Doppler frequencies uniformly distributed over [−0.5,−0.1], representing target leakage not masked by the main clutter.

Clutter-Region Contaminants (Sea Spikes): Interferences with SCNR = 0 dB and Doppler frequencies concentrated in the interval [0.1,0.4] around the clutter centroid, simulating high-power sea spikes or weak targets embedded in the main clutter lobe.

- Comparison Algorithms and Performance Metrics

The proposed IWP-ANMF (utilizing joint TLD-DSF features and adaptive weighting) is evaluated against two classical baselines: the Classical ANMF (using the unpurified Sample Covariance Matrix, SCM) and the GIP-based ANMF (which discards the 20% of samples with the largest Generalized Inner Product values and relies on the remaining data).

The evaluation is conducted using $10^{5}$ Monte Carlo trials across the following quantitative metrics:

Detection Probability vs. SCNR ($P_{d}$-SCNR): Target SCNR is varied from −10 dB to 15 dB in steps of 2 dB to measure absolute detection sensitivity.

Detection Probability vs. Doppler ($P_{d}$-$f_{d}$): Fixing target SCNR = 0 dB, the target velocity is swept from −0.5 to 0.5 to evaluate detection capability against spectral masking.

Normalized Mean-Square Error (NMSE): The estimation accuracy is rigorously evaluated using $\mathrm{NMSE}=∥\hat{R}-R_{true}{∥}_{F}^{2}/∥R_{true}{∥}_{F}^{2}$, where $R_{true}$ is the ideal covariance matrix derived from uncontaminated samples.

Auxiliary Metrics: Evolution of the effective sample size ($r_{eff}$), convergence iteration distributions, and practical deviations from the design CFAR value.

#### 4.1.2. Experimental Results and Discussion

**Figure 5:**
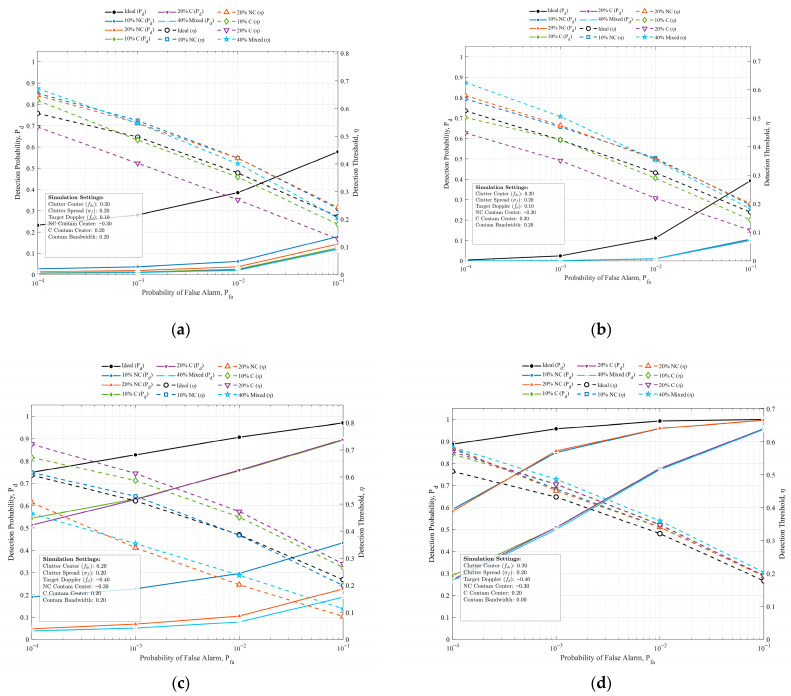
Comparative analysis of detection ROC curves and threshold variation curves across various contamination scenarios. (**a**) Target located in the clutter-dominated region and v=0.5. (**b**) Target located in the clutter-dominated region and v=10. (**c**) Target located in the noise-dominated region and v=0.5. (**d**) Target located in the noise-dominated region with a non-overlapping interference bandwidth and v=10.

Figure 5 illustrates a comparative analysis of the Receiver Operating Characteristic (ROC) curves and detection threshold variation curves derived from typical clutter simulations across varying target contamination ratios, sea states (v), and target Doppler locations. Based on the quantitative analysis, the following four critical regularities and characteristics can be deduced:

First, contamination-induced mismatch leads to monotonic degradation and threshold depression. Sample contamination induces a severe covariance matrix mismatch, resulting in a significant degradation of detection probability ($P_{d}$) under constant false alarm rate ($P_{fa}$) constraints. This deterioration exhibits a dose-dependent mechanism, wherein higher contamination ratios yield more severe performance penalties. Simultaneously, the contaminated covariance matrix estimate ($\hat{R}$) forces a downward shift in the relative detection threshold (η). Increased contamination severity correlates with a more pronounced threshold reduction, which compresses the detector’s discriminative capability between real targets and false alarms.

Second, severe sea states amplify contamination sensitivity in clutter-region detection. In the context of target detection within the clutter-dominated region (comparing Figure 5a,b), the severity of the sea state significantly impacts detection robustness. Under higher sea conditions (i.e., a smaller K-distribution shape parameter, v=0.5), the degradation in detection performance across different contamination ratios becomes notably more pronounced. This indicates that heavy-tailed clutter backgrounds in high sea states render the covariance estimation far more vulnerable to anomalous contamination compared to calmer sea states (v=10).

Third, the target-Doppler overlapping effect is substantially more destructive in the noise region. Whether the contamination samples overlap with the target’s Doppler frequency plays a decisive role in performance degradation. Contamination containing the target Doppler induces a drastic drop in detectability. Notably, this overlapping impact exhibits regional asymmetry: it exerts a much more devastating effect when the target is located in the noise-dominated region (as seen in Figure 5c) than when it is embedded in the clutter-dominated region.

Finally, spectral decoupling effectively preserves detection robustness. As an additional observation derived from the non-overlapping interference scenario (Figure 5d), when the contamination bandwidth in the noise region completely excludes the target’s Doppler frequency, the detection probability exhibits exceptional robustness, closely approximating the ideal uncontaminated upper bound. This phenomenon demonstrates that the “target masking effect” is fundamentally driven by localized spectral competition in the specific Doppler bins, rather than merely global covariance matrix distortion.

**Figure 6:**
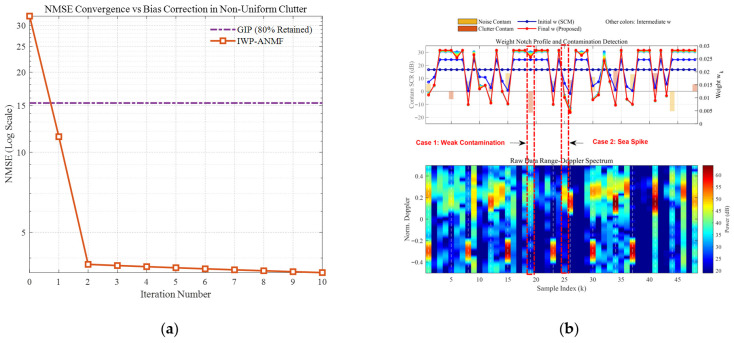
Convergence curve of NMSE with respect to iterations (v=0.5). (**a**) the logarithmic convergence curve of the Normalized Mean-Square Error (NMSE) versus iterations; (**b**) the weight notch evolution profile alongside the contamination distribution (**top right**), and the raw Range-Doppler spectrum (**bottom right**).

**Figure 7:**
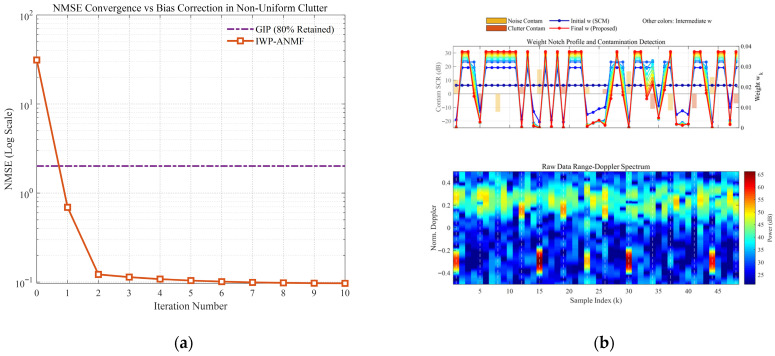
Convergence curve of NMSE with respect to iterations (v=10). (**a**) the logarithmic convergence curve of the Normalized Mean-Square Error (NMSE) versus iterations; (**b**) the weight notch evolution profile alongside the contamination distribution (**top right**), and the raw Range-Doppler spectrum (**bottom right**).

Figure 6 and Figure 7 comprehensively illustrate the dynamic convergence profile of the proposed algorithm when processing heterogeneous contaminated samples under two typical sea states (high sea state v=0.5 and low sea state v=10). Each figure comprises the logarithmic convergence curve of the Normalized Mean-Square Error (NMSE) versus iterations (left), the weight notch evolution profile alongside the contamination distribution (top right), and the raw Range-Doppler spectrum (bottom right). A profound comparative analysis reveals the following core mechanisms and regularities:

1. Coarse-to-Fine “L-shaped” Convergence Dynamics Aligned with Algorithmic Design. Observing the NMSE evolution curves, the estimation error exhibits a highly consistent “L-shaped” convergence trajectory. In the initial phase (the first 1–2 iterations), the NMSE experiences a precipitous drop, corresponding to the “coarse screening” stage with a large step size, which rapidly strips away high-energy, obvious outliers. Subsequently, the curve transitions into a gradual asymptotic stabilization plateau, reflecting the “fine-tuning” stage with a smaller step size. This “fast-then-slow, coarse-to-fine” dynamic behavior perfectly aligns with the two-phase decay adaptive step-size strategy designed in this paper.
2. Sequential Weight Suppression with Temporal Depth and Robustness. The Weight Notch Profile (top right) intuitively unveils the algorithm’s perceptual mechanism. For strong contaminated samples located in the noise region with a high Signal-to-Clutter-plus-Noise Ratio (SCNR), the algorithm achieves instantaneous identification during the initial iterations, assigning them infinitesimally small weights (manifested as deep notches). More importantly, as iterations deepen and the covariance matrix progressively purifies, the algorithm demonstrates fine-grained searching capabilities to capture heavily masked anomalies, effectively demonstrating the robustness of the proposed weighting function. Specifically, for typical heavy-tailed sea spikes (simulated via low-shape-parameter models) that exhibit high Target-Likeness but low Doppler Sharpness, the joint function actively captures them and progressively assigns sufficiently low weights in subsequent iterations (see the dashed boxes in Figure 6b). Furthermore, for ultra-low SNR (–15 dB) anomalies, rather than applying a rigid binary cut-off, the soft-weighting mechanism applies a moderate penalty proportional to the spike energy ratio. This temporal and adaptive penalization achieves thorough anomaly isolation, effectively preventing SCM contamination while optimally preserving the degrees of freedom from the remaining pure clutter components.
3. Exceptional Sea State Adaptability and Overwhelming Superiority over GIP. Comparing the different sea state conditions, the proposed IWP-ANMF achieves drastic improvements in estimation accuracy in both scenarios. However, its superiority over the traditional GIP method is particularly striking under the high sea state (Figure 6, v=0.5). In severe sea states characterized by heavy-tailed distributions and dense sea spikes, the GIP algorithm lacks multi-dimensional feature discrimination capability, misclassifying numerous sea spikes as safe samples. Consequently, its NMSE stagnates at a high-error plateau of approximately 15. Conversely, IWP-ANMF powerfully penetrates the sea spike interference, driving the NMSE down to a minimal value. This substantiates the irreplaceability of the joint feature space in harsh maritime environments.
4. Environment-Agnostic Convergence Rate and Evolutionary Stability. Beyond the aforementioned traits, the figures expose two crucial robustness characteristics. First, although v=0.5 and v=10 represent two physical environments with drastically divergent variance spans, the algorithm invariably reaches a steady state around the 3rd to 4th iteration in both scenarios. This indicates that the convergence rate of the algorithm is highly environment-agnostic. Second, tracking the weight connecting lines reveals that the sample weights exhibit a smooth, monotonically decreasing trend during the iterations, devoid of any numerical oscillatory rebounds. This implicitly corroborates the outstanding efficacy of the introduced matrix condition number regularization and smooth update mechanisms in maintaining system numerical stability.

**Figure 8:**
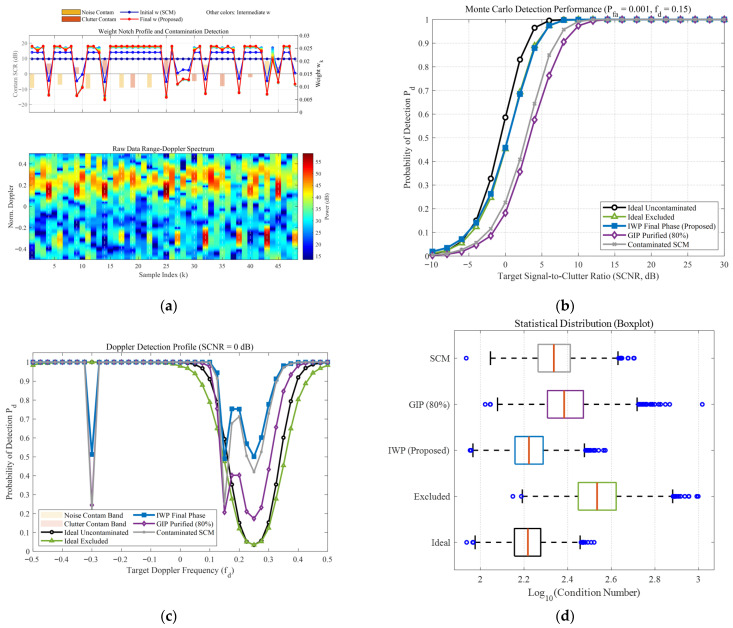
Comprehensive performance evaluation of the proposed IWP-ANMF algorithm under heterogeneous contamination scenarios. (Simulation parameters: number of pulses N=16, reference cells K=48, K-distribution shape parameter v=10, contamination ratio $P_{c}=40\%$, contamination SCR distribution: [−10, 10] dB, Doppler band of clutter-region contaminants: 0 dB, Doppler band of noise-region contaminants: 0 dB, and false alarm rate $P_{fa}=10^{-3}$). (**a**) Single-snapshot weight notch profile and raw Range-Doppler spectrum. (**b**) Probability of detection ($P_{d}$) versus target Signal-to-Clutter-plus-Noise Ratio (SCNR). (**c**) Doppler detection profile at a fixed SCNR = 0 dB. (**d**) Statistical distribution boxplot of the covariance matrix condition numbers (κ).

**Figure 9:**
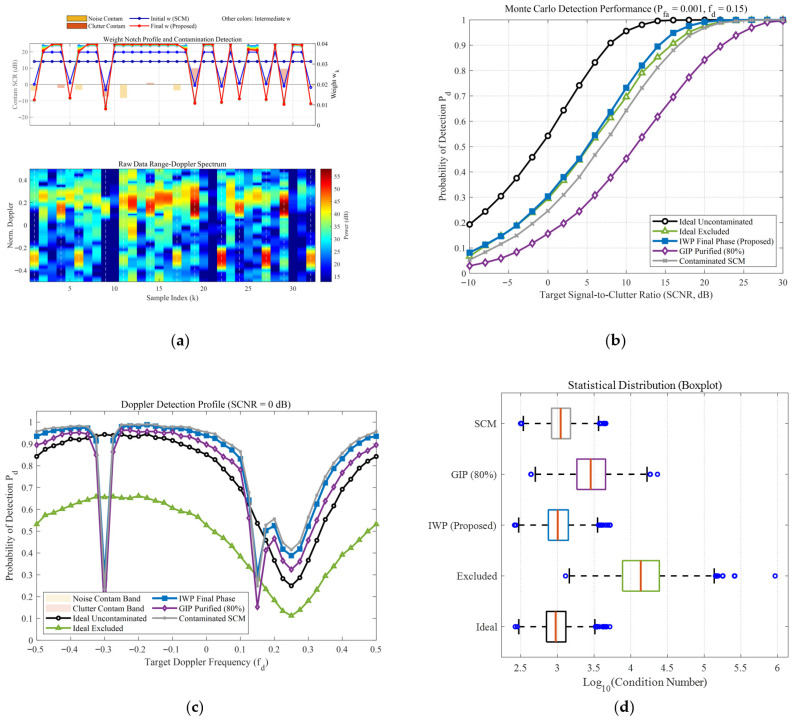
Comprehensive performance evaluation of the proposed IWP-ANMF algorithm under heterogeneous contamination scenarios. (Simulation parameters: number of pulses N=16, reference cells K=32, K-distribution shape parameter v=10, contamination ratio $P_{c}=40\%$, contamination SCR distribution: [−10, 10] dB, Doppler band of clutter-region contaminants: 0 dB, Doppler band of noise-region contaminants: 0 dB, and false alarm rate $P_{fa}=10^{-3}$). (**a**) Single-snapshot weight notch profile and raw Range-Doppler spectrum. (**b**) Probability of detection ($P_{d}$) versus target Signal-to-Clutter-plus-Noise Ratio (SCNR). (**c**) Doppler detection profile at a fixed SCNR = 0 dB. (**d**) Statistical distribution boxplot of the covariance matrix condition numbers (κ).

**Figure 10:**
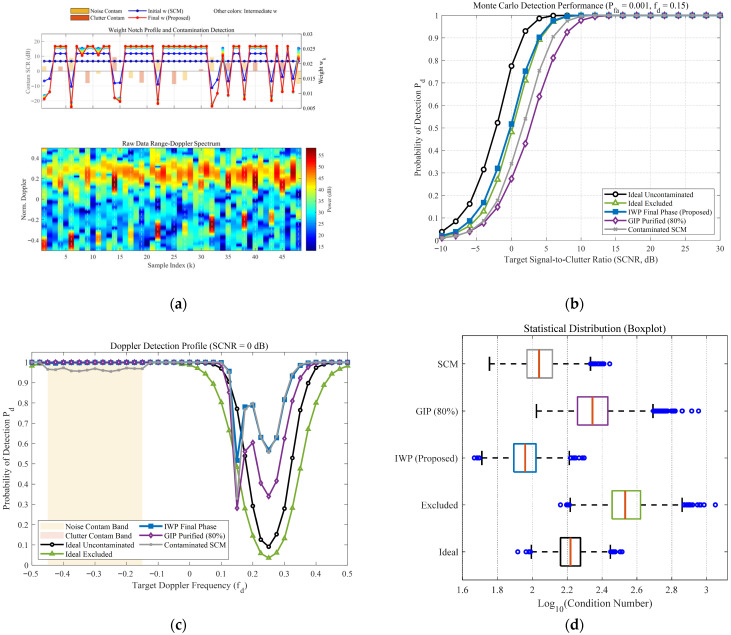
Comprehensive performance evaluation of the proposed IWP-ANMF algorithm under heterogeneous contamination scenarios. (Simulation parameters: number of pulses N=16, reference cells K=48, K-distribution shape parameter v=10, contamination ratio $P_{c}=40\%$, contamination SCR distribution: [−10, 10] dB, Doppler band of clutter-region contaminants: [−0.45, −0.15] dB, Doppler band of noise-region contaminants: 0 dB, and false alarm rate $P_{fa}=10^{-3}$). (**a**) Single-snapshot weight notch profile and raw Range-Doppler spectrum. (**b**) Probability of detection ($P_{d}$) versus target Signal-to-Clutter-plus-Noise Ratio (SCNR). (**c**) Doppler detection profile at a fixed SCNR = 0 dB. (**d**) Statistical distribution boxplot of the covariance matrix condition numbers (κ).

**Figure 11:**
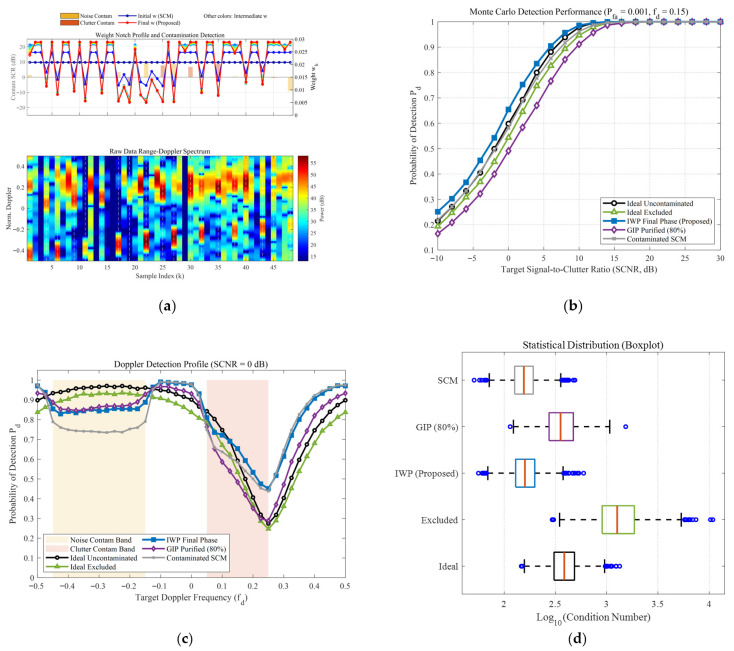
Comprehensive performance evaluation of the proposed IWP-ANMF algorithm under heterogeneous contamination scenarios. (Simulation parameters: number of pulses N=16, reference cells K=48, K-distribution shape parameter v=0.5, contamination ratio $P_{c}=40\%$, contamination SCR distribution: [−10, 10] dB, Doppler band of clutter-region contaminants: [−0.45, −0.15] dB, Doppler band of noise-region contaminants: [0.05,0.25], and false alarm rate $P_{fa}=10^{-3}$). (**a**) Single-snapshot weight notch profile and raw Range-Doppler spectrum. (**b**) Probability of detection ($P_{d}$) versus target Signal-to-Clutter-plus-Noise Ratio (SCNR). (**c**) Doppler detection profile at a fixed SCNR = 0 dB; (**d**) Statistical distribution boxplot of the covariance matrix condition numbers (κ).

To comprehensively evaluate the robustness of the proposed IWP-ANMF algorithm in complex heterogeneous environments, a multi-dimensional cross-validation framework was designed. This framework encompasses a high sea state reflecting severe heavy-tailed characteristics (v=0.5) and a low sea state approaching a Gaussian distribution (v=10), while introducing three progressive anomalous interference modes: discrete zero-bandwidth point contamination, noise-region band contamination, and combined dual-band contamination. Additionally, an extreme scenario with restricted secondary data (K=32) is incorporated. This multivariate testing architecture provides a rigorous closed-loop demonstration of algorithm performance, ranging from microscopic feature perception and macroscopic statistical detection to systematic numerical stability, as depicted in Figure 8, Figure 9, Figure 10 and Figure 11.

From the microscopic perspective of weight allocation and contamination perception (e.g., subplot (a) of the Figure 8, Figure 9, Figure 10 and Figure 11), the traditional Sample Covariance Matrix (SCM) assigns uniform initial weights to all samples due to the absence of a data-screening mechanism, making it highly vulnerable to anomalous data. Conversely, the proposed IWP mechanism demonstrates high-resolution physical discrimination capabilities. In the simulations, whether facing discrete isolated sea spikes or wideband combined contamination covering continuous Doppler intervals, the algorithm adaptively forms prominent “weight notches” at the contaminated sample indices during the final iteration stage. This precise weight suppression indicates that even in severe band-contamination scenarios where target signals and clutter spectra are deeply intertwined, the algorithm maintains fine-grained anomaly isolation efficacy.

Regarding macroscopic statistical detection performance, the absolute detection sensitivity (subplot (b) of the Figure 8, Figure 9, Figure 10 and Figure 11) and Doppler-domain anti-masking capability (subplot (c) of the Figure 8, Figure 9, Figure 10 and Figure 11) further quantify the algorithmic differences under varying sea states, bandwidth conditions, and sample sizes. Under the low sea state (v=10), conventional algorithms can still maintain baseline detection performance; however, when the environment degrades to the high sea state (v=0.5), the superposition of heavy-tailed characteristics and broadband contamination induces severe covariance matrix distortion in the SCM, leading to a precipitous drop in detection probability. Notably, the impact of limited sample support is explicitly evaluated in Figure 9 (K=32). In this extreme scenario, due to the high proportion of contamination, the effective number of pure samples falls well below the theoretical breakdown threshold of covariance matrix estimation (i.e., Reed’s rule of 2N, where N=16). The dual constraints of sample scarcity and heterogeneous interference drastically magnify the performance disparities among the algorithms, causing a severe performance collapse in the traditional SCM and GIP methods. In contrast, the proposed IWP algorithm resiliently mitigates this degradation, maintaining a detection curve that closely approximates the sub-optimal upper bound of the “Ideal Excluded” condition. Furthermore, when confronted with continuous broadband contamination, the clutter notch resulting from the GIP mechanism is severely broadened, which significantly narrows the usable Doppler bandwidth and makes targets highly susceptible to masking at the band edges (as shown in the shaded regions of subplot (c) of the Figure 10 and Figure 11). The proposed algorithm not only ensures high-probability detection in non-interference regions but also effectively suppresses the clutter notch widening, exhibiting highly resilient anti-masking detection capabilities at the edges of the blind speed zone.

Finally, analyzing the distribution of matrix numerical stability and condition numbers (subplot (d) of the Figure 8, Figure 9, Figure 10 and Figure 11) from a linear algebraic perspective verifies the algorithm’s numerical reliability in engineering applications. Particularly under the complex sea state of v=0.5 and the limited sample condition of K=32, the extensive inclusion of high-power sea spikes causes the condition number of the SCM to surge by orders of magnitude, presenting an extreme right-skewed outlier distribution. In practical radar signal processing, this poses a substantial ill-conditioned risk during matrix inversion. Thanks to the synergistic effect of joint feature adaptive weighting and the diagonal loading regularization mechanism, the condition number distribution of the IWP algorithm exhibits excellent compactness. This mechanism not only stably eliminates extreme outliers across all contamination scenarios, but its median value also demonstrates remarkably low sensitivity to sea state fluctuations and interference bandwidths, frequently outperforming even the uncontaminated ideal state. This provides a reliable numerical guarantee for real-time and robust matrix inversion under dynamic and variable maritime conditions.

### 4.2. Measured Data Experiment

**Figure 12:**
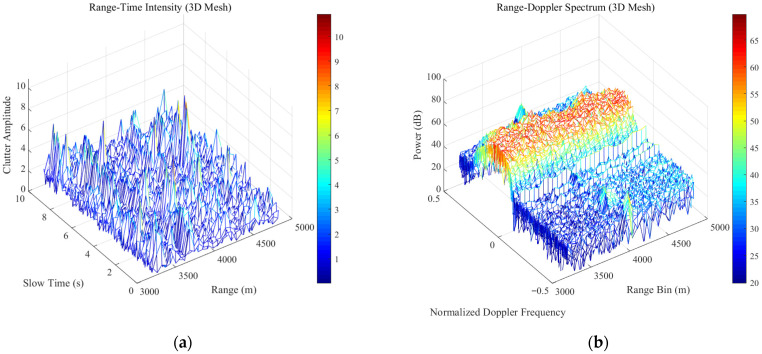
Data format of 19980204_223753_ANTSTEP.CDF (**a**) Time-range (range-pulse) two-dimensional intensity map; (**b**) range-gate-frequency (range-Doppler) two-dimensional spectrum.

**Figure 13:**
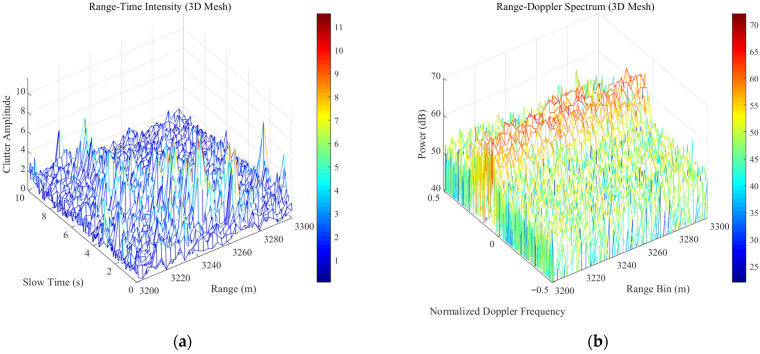
Data format of 19980223_185157_ANTSTEP.CDF: (**a**) Time-range (range-pulse) two-dimensional intensity map; (**b**) range-gate-frequency (range-Doppler) two-dimensional spectrum.

**Figure 14:**
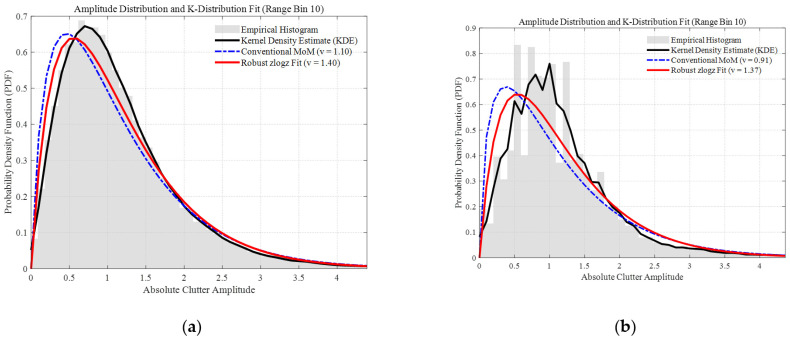
Amplitude probability density functions (PDF) and K-distribution fitting results at the 10th range bin for the two measured IPIX datasets. (**a**) Dataset 1 (19980204_223753_ANTSTEP.CDF); (**b**) Dataset 2 (19980223_185157_ANTSTEP.CDF). The figures illustrate the comparison between empirical histograms, Kernel Density Estimates (KDE), and the K-distribution fits using the Method of Moments (MoM) and the robust zlogz algorithm.

To comprehensively evaluate the robustness of the proposed IWP-ANMF algorithm under real-world maritime environments with varying radar parameters, two sets of typical sea clutter data from the IPIX radar database [33,34] with different range resolutions (60 m and 3 m) are employed in this study. The detailed system parameters for Dataset 1 (19980204_223753_ANTSTEP.CDF) and Dataset 2 (19980223_185157_ANTSTEP.CDF) are summarized in Table 10. The range-time (pulse) intensity characteristics and range-Doppler spectra of these two datasets are illustrated in Figure 12 and Figure 13, respectively. Furthermore, to quantitatively characterize the heavy-tailed severity of the measured clutter, the shape parameter v of the K-distribution for both datasets was estimated. The amplitude probability density distributions and the corresponding fitting results are detailed in Figure 14.

In the algorithm validation phase, unlike conventional simulations using purely synthetic data, this section constructs a massive empirical sample pool utilizing the aforementioned measured data (comprising 60,000 coherent pulses across valid range bins). During each Monte Carlo trial, sequential pulse snapshots are randomly extracted from the sample pool to dynamically formulate the Cell Under Test (CUT) with N pulses and the K secondary reference cells. To rigorously test the anti-interference capability of the algorithm, a specific proportion of heterogeneous contamination (encompassing target contamination in the noise region and sea spike contamination in the clutter region) is injected into the reference samples. Subsequently, target signals with varying Signal-to-Clutter-plus-Noise Ratios (SCNR) and Doppler frequencies are superimposed onto the CUT, thereby facilitating a full-chain evaluation and comparative analysis of target detection performance against a realistic clutter background.

**Figure 15:**
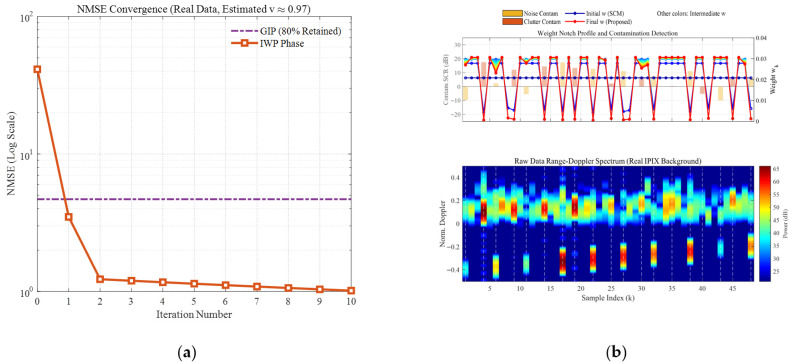

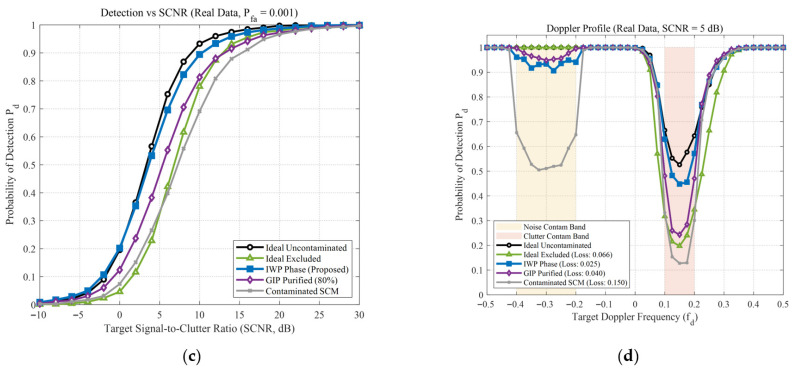
Full-chain detection performance evaluation using the measured IPIX Dataset 1 (19980204_223753_ANTSTEP.CDF, v≈0.97). (**a**) NMSE convergence curves versus iterations (using the covariance of the entire pure sample pool as the approximate ground truth). (**b**) Single-snapshot weight notch profile and Range-Doppler spectrum with the real clutter background. (**c**) Probability of detection ($P_{d}$) versus target SCNR ($P_{fa}=10^{-3}$). (**d**) Doppler detection profile at a fixed SCNR = 5 dB.

**Figure 16:**
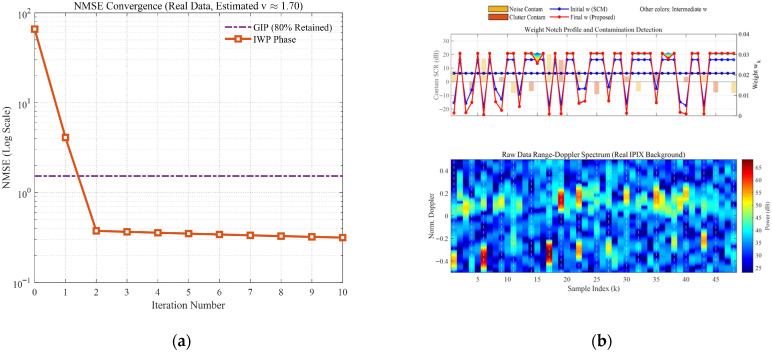

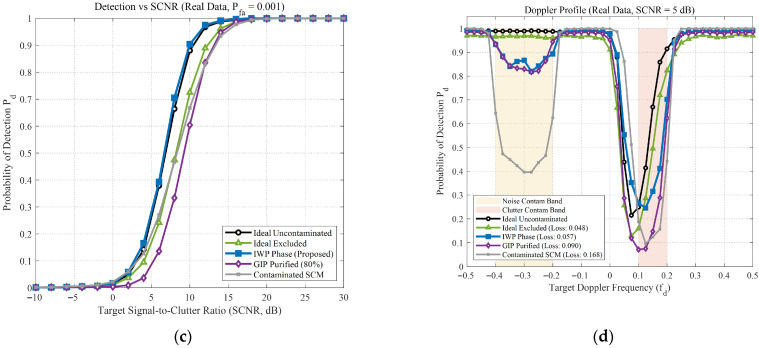
Full-chain detection performance evaluation using the measured IPIX Dataset 2 (19980223_185157_ANTSTEP.CDF, v≈1.70). (**a**) NMSE convergence curves. (**b**) Weight notch profile and Range-Doppler spectrum. (**c**) $P_{d}$ versus target SCNR. (**d**) Doppler detection profile.

Based on the full-chain Monte Carlo validations using the two measured IPIX datasets with different resolutions and sea state backgrounds under multi-band heterogeneous contamination, the following critical regularities and conclusions can be deduced:

1. Validity of Ground Truth Approximation and NMSE Convergence: In the evaluation utilizing measured data, since the true local covariance matrix is inaccessible, this section innovatively adopts the covariance matrix of the uncontaminated “entire empirical sample pool” as the approximate ground truth for calculating the Normalized Mean-Square Error (NMSE). As illustrated in Figure 15a and Figure 16a, even against a background characterized by real and complex internal correlations, the proposed algorithm achieves rapid NMSE convergence within 2 to 3 iterations. Its steady-state error is substantially lower than that of the traditional GIP algorithm, demonstrating the mechanism’s robust structure-recovery capability in authentic physical environments.
2. Asymmetry in Suppressing Noise-Region vs. Clutter-Region Contamination: Observations from Figure 15d and Figure 16d reveal differing algorithmic sensitivities to contamination across different frequency bands. Because measured sea clutter energy is predominantly concentrated in the low-frequency mainlobe, clutter-region contamination (e.g., sea spikes) often masks itself within the authentic background clutter, making it difficult for traditional methods to distinguish. Conversely, noise-region contamination (e.g., wideband cluster targets) exhibits higher prominence relative to the local noise floor, which readily causes severe inflation of local eigenvalues in traditional SCM and GIP, leading to broader “clutter notch widening.” Through fine-grained discrimination in the joint feature space, the proposed algorithm effectively applies weight notches to contamination in both regions (see Figure 15b), particularly mitigating the invalid bandwidth loss in the noise region.
3. Impact of Sea State Parameter (v) and Resolution on Robustness: Comparing Dataset 1 (60 m resolution, v≈0.97), which exhibits prominent heavy-tailed phenomena, with Dataset 2 (3 m resolution, v≈1.70), which possesses a weaker tail, it is evident that the performance degradation of traditional algorithms is highly correlated with the v value. Under the severe environment of v≈0.97, the detection performance of GIP and SCM deteriorates significantly (Figure 15c). In contrast, the detection curves of the proposed algorithm consistently and closely approximate the “Ideal Excluded” upper bound in both datasets. This indicates that the robustness of the proposed algorithm possesses excellent immunity to fluctuations in radar range resolution and clutter shape parameters.
4. Enhanced Masking Resistance at the Edges of the Doppler Blind Zone: In the quantitative integral evaluation across the Doppler domain (comparison of Loss areas), wideband spectral contamination induces a severe “Masking Effect” in traditional detectors near the edges of the interference bands. While maintaining high detection rates in non-interference regions, the proposed algorithm minimizes the Doppler detection loss near the interference bands (e.g., in Figure 15d, the Loss for IWP is only 0.025, significantly lower than the 0.040 for GIP). This verifies that the algorithm can maximize the usable Doppler bandwidth of the system under measured wideband interference scenarios.

## 5. Conclusions

This paper addresses the challenging problem of radar weak target detection in nonhomogeneous sea clutter environments by proposing a novel adaptive detection algorithm based on iterative weighting and sample purification (IWP-ANMF). The proposed methodology establishes an innovative closed-loop feedback mechanism between detection and estimation, enabling the autonomous identification and adaptive suppression of anomalous samples within the reference window. Theoretically, the algorithmic procedure is rigorously formulated as a robust weighted covariance matrix estimation problem, with explicit derivations provided for its convergence conditions. Extensive full-chain simulation results demonstrate that, compared with conventional adaptive processing methods, the proposed algorithm achieves substantial robustness gains in scenarios contaminated by dense targets or severe sea spikes, effectively enhancing the probability of detection under low Signal-to-Clutter-plus-Noise Ratio (SCNR) conditions.

Nevertheless, the computational burden currently remains a limiting factor. The requirement for multiple covariance matrix estimations and matrix inversions during the iterative weighting and resampling processes elevates the overall computational complexity to $O(T\cdot K\cdot N^{3})$ (where T denotes the number of iterations). To a certain extent, this constrains the real-time processing capabilities of the system when handling extremely large-scale data. In summary, this study provides a theoretically grounded and practically feasible technical solution for robust adaptive processing in maritime radar applications, offering valuable insights and engineering references for enhancing detection performance under complex sea state conditions.

## Figures and Tables

**Table 1:** Qualitative comparison of the proposed method with existing SCM estimation approaches.

Method Category	Representative Approaches	Dependency on External Prior Knowledge	Adaptivity to Dynamic Environments	Handling of Random Sea Spikes	Computational Complexity
Statistical-driven	GIP, M-estimators	None	High	Limited (struggles with heavy-tailed spikes)	Low to Moderate
Knowledge-Aided (KA)	Clutter-map KA, GIS-based KA	High (Requires external databases)	Limited by database update rate	Dependent on historical records	High (Requires database querying)
Physics-informed (Proposed)	2D Joint Feature Soft-weighting	None (Relies strictly on echo data)	High (Real-time dynamic sensing)	Excellent (Joint suppression via target-likeness & Doppler sharpness)	Moderate (Iterative calculation)

**Table 2:** Correspondence between K-distribution shape parameter and sea state levels.

Shape Param.	Sea State	Sig. Wave Ht. (m)	Sea Surface Characteristics	Clutter Properties
0.3–0.8	6–7 (High)	>4	Large breaking waves, extensive whitecaps	Extreme spikiness, heavy tails
0.8–1.5	4–5 (Moderate)	2–4	Developing waves, local breaking	Moderate spikiness, non-Gaussian
1.5–3.0	2–3 (Low)	1–2	Small ripples, no breaking	Light tails, near-Rayleigh
>3.0	0–1 (Calm)	<1	Specular reflection dominant	Approximately Rayleigh

**Table 3:** Classification and characteristics of sea spikes.

Type	Physical Mechanism	Duration	Doppler Characteristics	Occurrence Conditions
Type A (Breaking)	Foam-covered region from wave breaking	0.1–1 s	Broad dispersion, bimodal	Wind > 7 m/s, wave breaking
Type B (Whitecap)	Specular reflection and multiple scattering from wind-induced whitecaps	0.5–3 s	Moderate broadening, center offset	Persistent wind fetch, developed whitecaps
Type C (Swell)	Coherent scattering from swell systems	1–10 s	Quasi-periodic, narrowband	Swell dominant, low wind area
Type D (Clutter)	Radar system noise or interference	Random	No specific structure	System malfunction or EMI

**Table 4:** Multi-dimensional characteristics of targets, sea spikes, and background clutter.

Feature Dimension	Real Target	Sea Spike	Background Clutter
Doppler Origin	Rigid-body translation, stable radial velocity	Wave breaking, random scatterer motion	Bragg scattering, surface micro-element motion
Spectral Peak Structure	Single peak, highly concentrated	Single/double peak, significantly broadened	Double peak (Bragg), dispersed
DSF Typical Range	0.7–1.0	0.4–0.6	0.1–0.3
Temporal Variation	Slow (target maneuver)	Fast (transient event)	Medium-slow (texture modulation)

**Table 5:** The comprehensive numerical stability monitoring and multi-tier regularization criteria implemented in the proposed algorithm.

Monitoring Metric	Threshold	Trigger Condition	Regularization Measure
Condition number κ(R)	$\kappa >\kappa_{max}$ (e.g., $10^{4}$)	Ill-conditioned matrix due to power disparity	CNC-ADL:R←R+γI, where $\gamma =max\left(0,\frac{\lambda_{max}-\kappa_{max}\lambda_{min}}{\kappa_{max}-1}\right)$
Minimum eigenvalue $\lambda_{min}$	$\lambda_{min}<\epsilon$ (e.g., $10^{-6}$)	Eigenvalue collapse/Machine precision limit	Eigenvalue truncation:$\lambda_{i}=max(\lambda_{i},\epsilon )$
Effective rank $r_{eff}$	$r_{eff}<N/2$	Excessive weight concentration (loss of DOF)	Tikhonov regularization/Weight smoothing

**Table 6:** Step-by-step execution procedure of the IWP-ANMF algorithm.

Step	Operation Description
Initialization	Initialize uniform weights $w_{k}^{\left(0\right)}=1/K$ and un-whitened matrix ${\hat{R}}^{\left(0\right)}=I.$
Step 1: Metric Extraction	Compute leave-one-out TLD spectra $\Lambda_{k}(f_{d})$ and structural DSF $\rho_{k}$ for each sample.
Step 2: Joint Feature	Calculate the joint discrimination feature $F_{k}=max(\Lambda_{k})\cdot \rho_{k}.$
Step 3: MAD Thresholding	Extract MAD(F) and establish the rigorous exclusion threshold $Th_{clean}.$
Step 4: Dynamic Update	Apply the two-phase decay step size ($\beta_{init}$ or $\beta_{iwp}$) to update weights $w_{k}^{\left(t+1\right)}.$
Step 5: Regularization	Normalize weights, perform recursive covariance update, and evaluate condition number.
Step 6: Convergence Check	Evaluate termination criteria ($\delta_{\Lambda },\rho_{w}$). If not met, return to Step 1.
Finalization	Output the purified robust covariance matrix ${\hat{R}}_{final}.$

**Table 7:** Correspondence between weight function morphology and influence function characteristics.

Sample Type	$\mathrm{Feature}\;(F_{k})$ Range	Weight Morphology	Implicit IF Characteristics
Background Clutter	Low	Approximately constant	Linear, full statistical efficiency
Boundary Region	Medium	Smoothly decelerating decay	Bounded, structural downweight protection
Outliers (Spikes/Jamming)	High	Exponential decay/truncation	Redescending, complete energy suppression

**Table 8:** Evolution of eigenvalue distribution during the iterative purification process.

Iteration Stage	Eigenvalue Distribution Characteristics	Physical Interpretation
t=0 (Initial)	Large eigenvalues dispersed, small values compressed, $\kappa >10^{10}$	Severe contamination, highly ill-conditioned estimate
t=1,2 (Coarse Phase)	Extreme eigenvalues forcefully suppressed	Rapid truncation of primary isolated outliers
t=3,4 (Fine Phase)	Eigenvalues tend to concentrate, effective rank stabilizes	Fine adjustment and sub-space purification
t≥5 (Convergence)	Compact distribution, approaching theoretical bounds	Robust clutter covariance estimation achieved

**Table 9:** Summary of experimental parameter configurations.

Parameter Category	Parameter Description	Value/Setting
Radar System	Number of coherent pulses (N)/System DOF	16
	Number of reference samples (K)	32,48
	Target normalized Doppler frequency ($f_{d}$)	0.15
	Design false alarm probability ($P_{fa}$)	$10^{-3}$
Sea Clutter Model	Distribution model	Compound-Gaussian (K-distribution)
	Shape parameter (ν)	0.5 (High sea state); 10 (Low sea state)
	Speckle correlation coefficient (ρ)	0.9
	Normalized Doppler centroid ($f_{dc}$)	0.1
Contamination	Contamination ratio ($P_{c}$)	10%,20%,40%
Scenario	Noise-region contaminants Doppler band	0.2 (Cluster targets)
	Clutter-region contaminants Doppler band	0.2 (Clutter spikes)
	Contaminant SCNR	[−10, 10] dB
	Target SCNR Range	[−10, 30] dB
	Fixed Target SCNR (Doppler Slice)	0 dB

**Table 10:** System parameters of the two sets of IPIX radar data used in the experiment.

Parameters	Dataset 1	Dataset 2
Filename	19980204_223753_ANTSTEP.CDF	19980223_185157_ANTSTEP.CDF
Date and time/UTC	4 February 1998 22:37:53	23 February 1998 18:51:57
RF frequency	9.39 GHz	9.39 GHz
Pulse length	400 ns	20 ns
Pulse repetition frequency	1000 Hz	1000 Hz
Range resolution	60 m	3 m
Radar azimuth angle	359.8° *	130.4° *
Antenna gain	45.7 dB	45.7 dB
Radar beamwidth	0.9°	0.9°

* Note: The radar azimuth angle may vary depending on the specific staring direction of the dataset.

## Data Availability

The data presented in this study are available on request from the corresponding author.
